# Sub-Retinal Injection of Human Lipofuscin in the Mouse - A Model of “Dry” Age-Related Macular Degeneration?

**DOI:** 10.14336/AD.2022.0626

**Published:** 2023-02-01

**Authors:** Nan Su, Uwe Hansen, Tanja Plagemann, Karin Gäher, M. Dominik Leclaire, Jeannette König, Annika Höhn, Tilman Grune, Constantin E. Uhlig, Nicole Eter, Peter Heiduschka

**Affiliations:** ^1^Research Laboratory, Department of Ophthalmology, University Medical Center, Münster, Germany.; ^2^Institute of Musculoskeletal Medicine, Medical Faculty, University of Münster, Münster, Germany.; ^3^German Institute of Human Nutrition, Potsdam-Rehbrücke, Germany.; ^4^Cornea Bank Münster, Department of Ophthalmology, University Medical Center, Münster, Germany.; ^5^Department of Ophthalmology, University Medical Center, Münster, Germany.; ^6^Department of Ophthalmology, The First Affiliated Hospital of Zhengzhou University, Zhengzhou, China.

**Keywords:** Lipofuscin, microglia, retinal pigment epithelium, age-related macular degeneration, electroretinography, electron microscopy

## Abstract

Lipofuscin (LF) accumulates during lifetime in the retinal pigment epithelium (RPE) and is thought to play a crucial role in intermediate and late age-related macular degeneration (AMD). In an attemt to simulate aged retina and to study response of retinal microglia and RPE cells to LF, we injected a suspension of LF into the subretinal space of adult mice. LF suspension was obtained from human donor eyes. Subretinal injection of PBS or sham injection served as a control. Eyes were inspected by autofluorescence and optical coherence tomography, by electroretinography and on histological and ultrastructural levels. Levels of cytokine mRNA were determined by quantitative PCR separately in the RPE/choroid complex and in the retina. After injection of LF, microglial cells migrated quickly into the subretinal space to close proximity to RPE cells and phagocytosed LF particles. Retinal function was affected only slightly by LF within the first two weeks. After longer time, RPE cells showed clear signs of melanin loss and degradation. Levels of mRNA of inflammatory cytokines increased sharply after injection of both PBS and LF and were higher in the RPE/choroid complex than in the retina and were slightly higher after LF injection. In conclusion, subretinal injection of LF causes an activation of microglial cells and their migration into subretinal space, enhanced expression of inflammatory cytokines and a gradual degradation of RPE cells. These features are found also in an aging retina, and subretinal injection of LF could be a model for intermediate and late AMD

Age-related macular degeneration (AMD) is the leading cause of irreversible legal blindness [1]. With the aging of the population, the incidence of AMD is very likely to gradually increase.

One possible player in pathogenesis of AMD is the “age pigment” lipofuscin (LF), a yellowish pigment consisting of a big variety of oxidised lipids and proteins [2]. It shows strong autofluorescence due to a high amount of A2E, an oxidative by-product of the visual cycle [3]. In the eye, LF accumulates in the cells of the retinal pigment epithelium (RPE) during the lifetime. It has been thought for many years that RPE cells cannot secrete accumulated LF. However, there are strong indications that RPE cells are able to release LF particles, mainly towards Bruch’s membrane [4], and also into the subretinal space (Thomas Ach, personal communication). This raises the question which effects such released LF may have in the posterior part of the eye. It may be taken for granted that RPE cells themselves show changes in their behaviour at higher ages due to an overload with LF, and it is also possible that RPE cells react when confronted with extracellular LF [5].

In addition, response of retinal microglia (MG) to extracellular LF could also contribute to pathologies in the back of the eye, as it is known that MG cells migrate into the subretinal space at higher age, in particular in the case of AMD [6-9]. In a previous study, we have shown that cultured MG cells produce many inflammatory cytokines when human LF isolated from donor eyes was added to the culture medium [10]. It thus may be supposed that MG cells show an inflammatory behaviour also in the living retina when getting in touch with extracellular LF.

To study consequences of extracellular LF in the back of the eye, we injected human LF isolated from donor eyes into the subretinal space of mice, and we performed various examinations regarding retinal morphology on both histological and ultrastructural levels, production of cytokines, and retinal function. This way, we want to establish an experimental animal model for age-related changes of the retina.

## MATERIALS AND METHODS

### Preparation of lipofuscin

**Figure 1.**
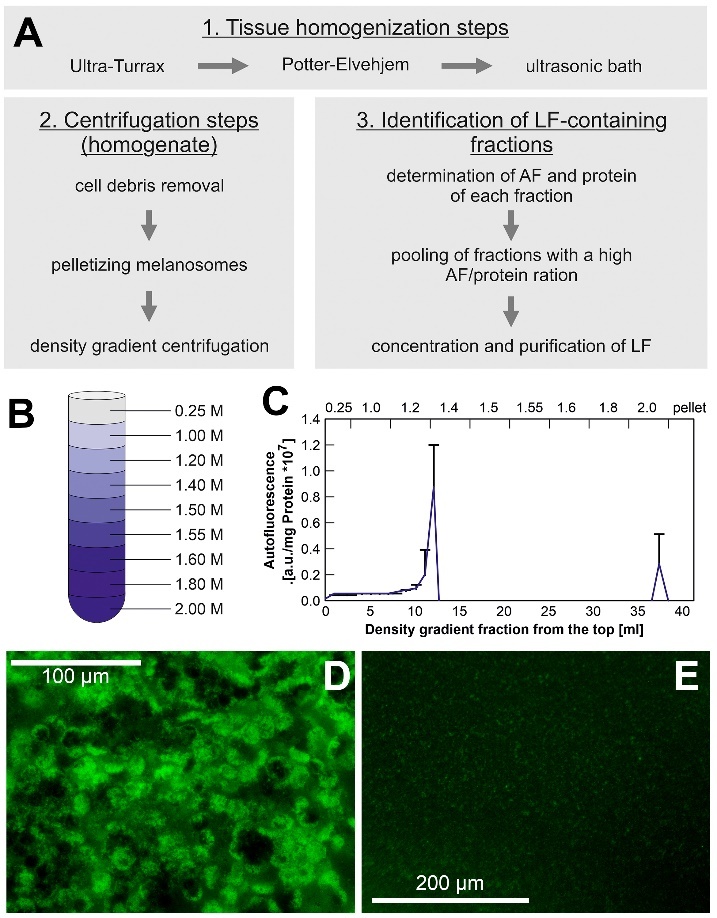
Preparation of human lipofuscin used in this study. (A) Flowchart of lipofuscin (LF) isolation. (B) Sucrose gradient used for separation of supernatant fractions. (C) Autofluorescence of fractions in relation to protein content. (D) Cells of the retinal pigment epithelium in a human donor eye. LF is visible by its strong autofluorescence. (E) Appearance of autofluorescent lipofuscin particles in the final suspension.

Lipofuscin (LF) was obtained from the RPE of human donor eyes. The eyes were provided by the cornea bank of the Department of Ophthalmology, University of Münster Medical Centre. The donor eyes were obtained from elderly donors and did not show signs of specific path-ological changes apart from those associated with aging. Written consent about the use of donor material for scientific purposes had been given by all donors or by the donor’s relatives according to the donor’s will. We isolated the RPE/choroid complex from more than 50 eyes and pooled the tissue before further processing. The procedure of LF preparation we applied is shown in Figure 1. It was described in detail in [10] and was based on an established procedure developed by Boulton & Marshall (1985) [11]. We used the one and the same LF suspension for all injections in this study.

### Animals

Wild-type C57BL/6J male and female mice were used for the present study, ranging between 4 and 6 months. All experiments were performed in accordance with the ARVO Statement for the Use of Animals in Ophthalmic and Vision Research and the EU directive 2010/63/EU. They were approved by the local authorities (LANUV, Recklinghausen, Germany, file number 84-02.04.2012. A063). Mice were held in ventilated cages at a 12 hours/12 hours light/dark cycle with standard food and drinking water ad libitum.

### Subretinal injection of lipofuscin suspensions

The animals were anaesthetised by inhalation anaesthesia with 3% isoflurane in oxygen. The eye of the mouse was rotated slightly. The conjunctiva was opened, and a sharp 30-gauge cannula was used to cut an opening in the sclera approximately half a millimetre behind the limbus. Through this, a blunt 33-gauge cannula was inserted tangentially to the globe and moved about 1.5 mm along the back of the eye as close as possible to the RPE/choroid complex. Thereafter, a volume of 1 μl of the lipofuscin suspension was injected. As lipofuscin is a mixture of different compounds, we used suspensions of the same fluorescence intensity (0.8 according to Figure 1C). As a control, a volume of 1 μl of sterile phosphate-buffered saline (PBS) was injected. After another three to four seconds, the cannula was pulled out slowly of the eye, and the eye was turned back to its original position. After the injection, mice were brought back into their cage and allowed to recover. In addition to the experimental groups where PBS or LF suspension were injected, we used a third group where just a sham injection was performed, *i.e.*, the surgical procedure was the same except that no liquid was injected into the eye. In total, LF suspension was injected in 48 mice, PBS was injected in 35 mice, and sham treatment was performed in 8 mice. Moreover, 8 mice were used as treatment-naïve controls.

### In vivo imaging

Mice were anaesthetised by an intraperitoneal injection of a mixture of 130 mg/kg ketamine and 2.7 mg/kg xylazine. Their pupils were fully dilated with 1% tropicamide and 5% neosynephrine, while the sleeping animals were placed on a heating pad. Mice were put in front of the "Spectralis" device from Heidelberg Engineering, and they were examined by infrared laser scanning ophthal-moscopy (IR-SLO), multicolour imaging and optical coherence tomography (OCT).

### Electroretinography

Mice were dark-adapted for at least 12 hours and prepared for measurement at dim red light the same way as for *in vivo* imaging. In addition, the cornea was desensitised with proparacaine. ERG measurement was performed using the RetiPort animal system from Roland Consult (Brandenburg, Germany). For the duration of the measurement, the animals were placed on a plate heated at 37°C to prevent cooling of the animals. Gold ring electrodes were placed on the cornea of the eyes without damaging the cornea. As the reference electrode, another gold electrode was used, which was moistened with saline and placed into the mouth of the animals.

Scotopic and photopic ERG were measured. Scotopic ERG was measured at six different light intensities of the flashes (0.0003, 0.003, 0.03, 0.3, 3 and 30 cd•s/m²). Isolated oscillatory potentials were recorded at 30 cd•s/m². Photopic ERG measurement was performed with a backlight of 25 cd/m² at four light intensities (1, 3, 10 and 100 cd•s/m²), isolated oscillatory potentials at 100 cd•s/m² and the 30 Hertz flicker ERG at 3 cd•s/m². In order to assess whether and to what extent injection of lipofuscin influences retinal function, the parameters of the different experimental groups were compared. For the evaluation, the amplitudes, and latencies of the a- and b-waves and the b/a ratio in the animals of the experimental groups were extracted from the measurements, as well as the amplitudes and latencies of the oscillatory potentials and the amplitudes of the 30-Hertz flicker ERG.

### Tissue processing, histology and fluorescent immunohistochemistry

Eyes were isolated at different time points after injections as indicated in the Results section. At least two females and two males were used in every group, and typical examples are shown. Eyes were isolated and fixed in 4% paraformaldehyde for 1 h, washed 2× in PBS pH 7.4 for 5 min and frozen in NEG-50™. Cryo sections (thickness 10 µm) and were cut using a Cryostar NX70 cryostat (Thermo Fisher Scientific), placed on Starfrost Advanced Adhesive glass slides (Engelbrecht) and were stored at -20 °C until used for histology and immunohistochemistry. For immunohistochemical staining of microglial cells, sections were blocked with Power Block™ reagent (HK085-5K, BioGenex) at room temperature for 6 min, then washed 3× with 0.1 M PBS and incubated overnight at 4°C with primary antibodies against Iba1 (from guinea pig, Synaptic Systems, #234003, 1:500) or CD11b (from rat, Serotec, #MCA711, 1:60). The sections were then washed 3×with 0.1 M PBS and incubated with appropriate secondary antibodies for 1 h at room temperature (anti-guinea pig, abcam, #ab150185, 1:400, or anti-rat, abcam, #ab6732, 1:200). The nuclei were counterstained with DAPI (4′6′-diamidino-2-phenylindole dihydrochloride) diluted with pure water 1:300 for 7 min at room temperature. Finally, sections were washed 3× with 0.1 M PBS and mounted under glass coverslip using mounting media (ImmuMount™, Thermo Scientific). The primary antibodies were diluted with 1% bovine serum albumin containing 0.1% Triton X-100, and secondary antibodies were diluted with 1% bovine serum albumin. We optimised dilutions of antibodies for best specific staining and lowest possible background fluorescence. So-called “negative controls” were performed for all antibodies, *i.e.*, staining procedures were performed where primary antibodies were omitted. Non-specific background staining was neglectable in every case. For digital imaging, an epifluorescence microscope (EVOS fl, Advanced Microscopy Group, USA) was used.

### Investigation of protein expression by quantitative PCR

Quantitative PCR was performed in animals injected with PBS or LF suspension, as well as in non-treated animals. Whole eyes were isolated and stored shortly in PBS on ice before further processing. The retina and the RPE-choroid complex were prepared separately and put into 3.5 μl β-mercarptoethanol in 350 μl lysis buffer. The samples were stored at -80°. Total RNA was purified from the samples follow routine procedures using the RNeasy® Mini Kit (50) (QIAGEN, cat. no. 74104) with QIAshredder (250) REF 79656 (QIAGEN) and RNase-free DNase Set (50) REF 79254 (QIAGEN). To obtain the cDNA, we used the iScript™ cDNA Synthesis Kit (BIO RAD), cat. no. 170-8891. The assays used in this study are listed in Table 1.

**Table 1:** Gene Expression Assays used in this study.

Gene Expression Assay	Order Number
Act-β (β-Actin) housekeeping gene	Mm02619580-g1
GAPDH housekeeping gene	Mm99999915-g1
CCL-2	Mm00441242-m1
FGF-2	Mm00433287-m1
IL-6	Mm01210733-m1
IL-1β	Mm99999061-mH
CXCL-1 (KC)	Mm04207460-m1
TNF-α	Mm99999068-m1

### Transmission electron microscopy

Eyes were isolated and immediately fixed in 2% (v/v) formaldehyde and 2.5% (v/v) glutaraldehyde in 100 mM cacodylate buffer, pH 7.4, at 4°C overnight. After washing in PBS, the eyes were post fixed in 0.5% (v/v) osmium tetroxide and 1% (w/v) potassium hexacyanoferrate (III) in 0.1 M cacodylate buffer for 2 h at 4°C followed by washing with distilled water. After de-hydration in an ascending ethanol series from 30% to 100% ethanol, specimens were incubated two times in propylenoxide each for 15 min and embedded in Epon. Ultrathin sections were cut with an ultramicrotome, collected on copper grids, and negatively stained with 2% uranyl acetate for 10 min. Electron micrographs were taken at 60 kV with a Phillips EM-410 electron micro-scope using imaging plates (Ditabis, Pforzheim, Germany).

### Statistics

Levels of significance were calculated, and graphs were prepared using Prism™ 9 software (GraphPad Software Inc., La Jolla, CA, USA). We decided *a priori* to use non-parametric statistics and did not check the data for normal distribution. Levels of significance were calculated using the Kruskal-Wallis test with Dunn's multiple comparisons test. P values of <0.05 were considered statistically significant.

**Figure 2.**
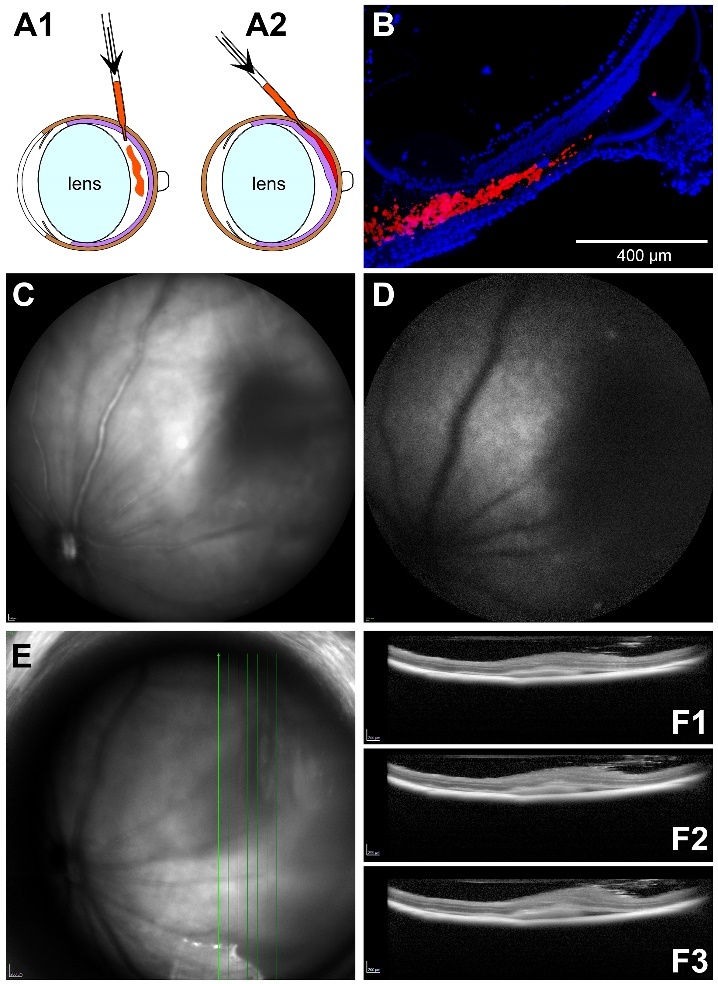
Principle of subretinal injection. (A) Comparison of intravitreal (A1) and subretinal (A2) injections. (B) Cryosection of a mouse eye after subretinal injection of fluorescent beads (red). Nuclei of the cells are labelled with DAPI (blue). Scale bar: 400 µm. (C) Infrared-SLO image and (D) autofluorescence image immediately after subretinal injection of lipofuscin suspension. (E) IR-SLO image and (F) three OCT b-scans 1 day after subretinal injection of LF.

## RESULTS

Subretinal injection (Fig. 2) could be performed success-fully in most of the mice. There was not a single case of endophthalmitis or similar inflammatory reaction in the eyes after subretinal injection. As described above, the needle of the syringe is inserted tangentially to the eyeball, which is in contrast to intravitreal injections (Figs. 2A1 and 2A2). To verify suitability of the applied technique, we first injected fluorescent microbeads and checked their location in freshly prepared cryosections. Injected microbeads were clearly visible in the subretinal space (Fig. 2B). To further check success of subretinal injection procedure, we inspected mouse eyes by *in vivo* imaging. In IR-SLO, a darkening was visible in the area of injection (Fig. 2C). In the autofluorescence (AF) image, an enhanced AF was visible in the vicinity of the dark area (Fig. 2D). It may be speculated that increased AF could be caused by injected LF, whereas the darkened area probably may indicate retinal detachment. OCT scans were performed 1 day after injection of LF through the dark area (Fig. 2E). In the b-scans, indeed a detachment of the retina was seen, with some opaque material between the retina and the RPE (Figs. 2F1-3).

**Figure 3.**
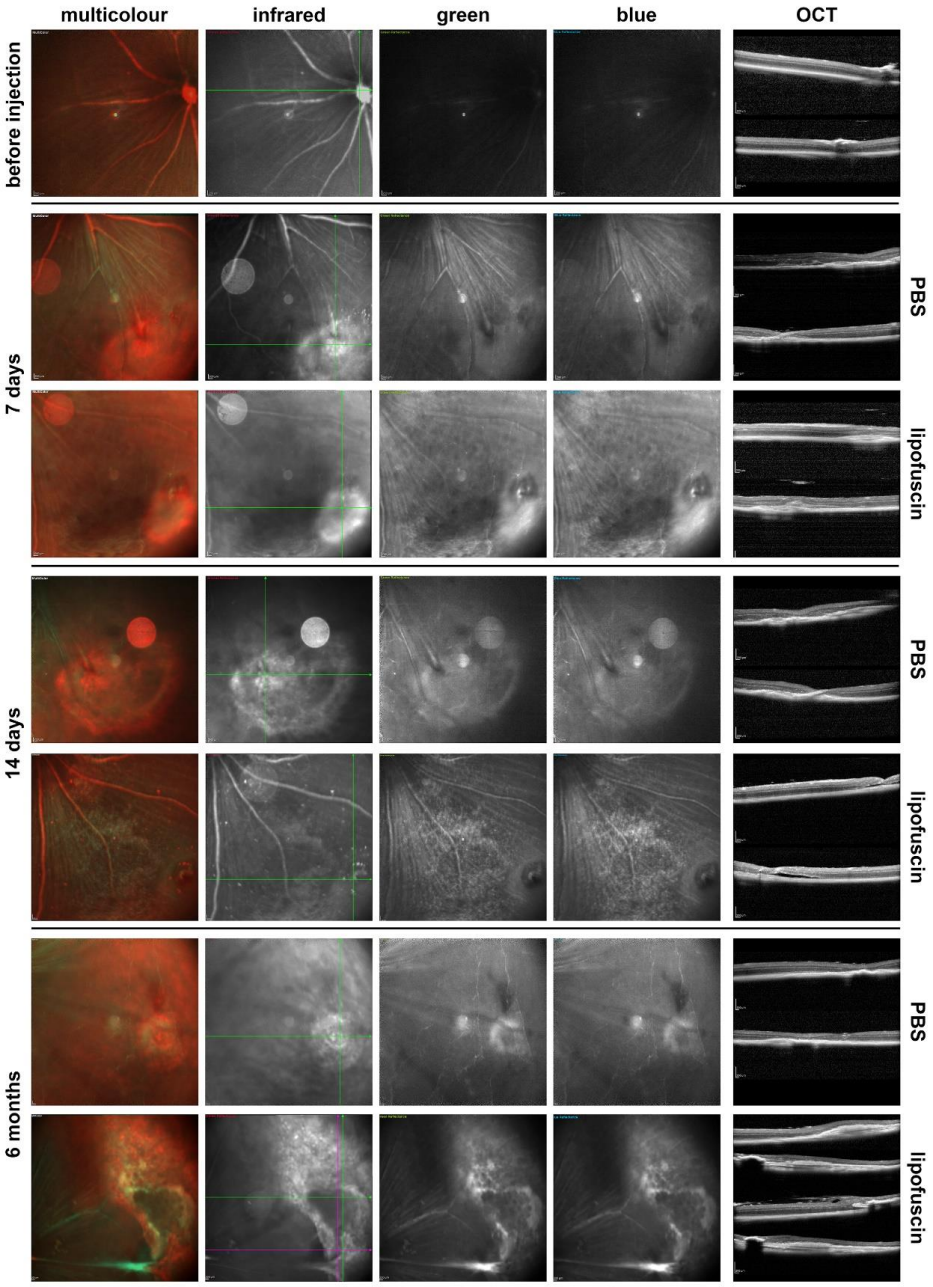
Multicolour imaging of eyes injected with PBS or LF. Images taken in the multicolour SLO mode of the injection site 7 days (n=6), 14 days (n=6) and 6 months (n=4) after subretinal injection of either PBS or LF as indicated. On the right side, OCT images are shown of b-scans taken as indicated by the lines in the infrared image (first horizontal, then vertical scans).

**Figure 4.**
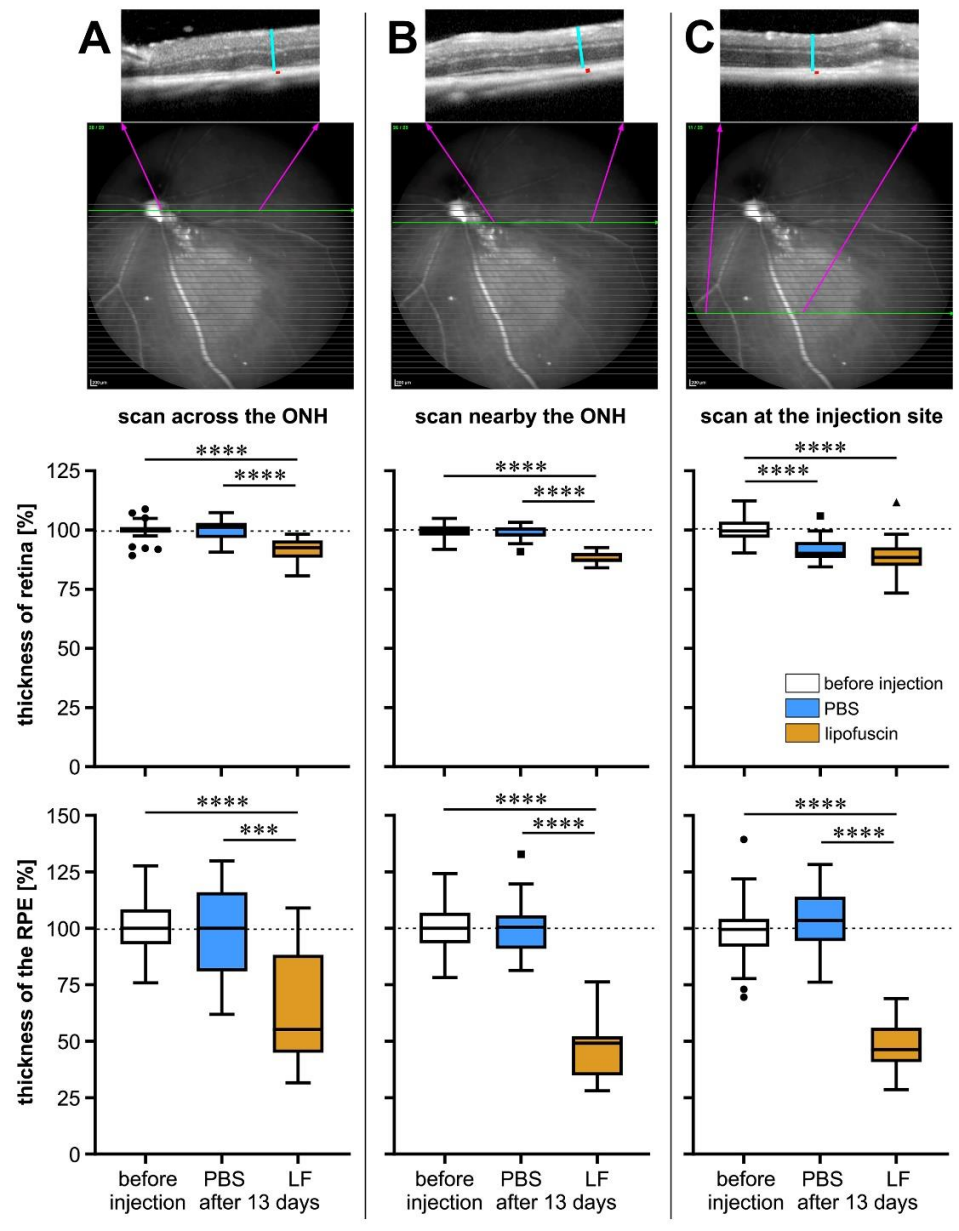
Measurement of retinal thickness and RPE layer thickness in mouse eyes. Measurement was performed before injection (n=43) and 13 days after injection of PBS (n=13) or LF (n=22) in OCT scans recorded at three different sites in each eye: (A) across the optic nerve head (ONH), (B) nearby the ONH, and (C) at the injection site. Values were determined as indicated on top, where turquois lines indicate retinal thickness and red dots RPE layer thickness. Data are presented as box plots after Tukey, and significance of differences is indicated by *** p<0.001 and **** p<0.0001, with p values calculated by the Kruskal-Wallis test with Dunn's multiple comparisons test.

After a subretinal injection, a certain tissue pro-liferation may occur around the site of injection in the subretinal space, which is visible after PBS injection as well as after LF injection in the infrared light in the SLO. However, an increased autofluorescence under excitation with blue or green light was stronger after subretinal injection of LF. Such autofluorescence may remain after a longer time, even after 6 months (Fig. 3). There are also stronger structural distortions of the retina after a subretinal injection of LF compared to injection of PBS, as can be seen in the OCT scans shown in Fig. 3. Besides retinal detachments, such distortions include more subretinal material and epiretinal membranes that become visible after six months (Fig. 3).

**Figure 5.**
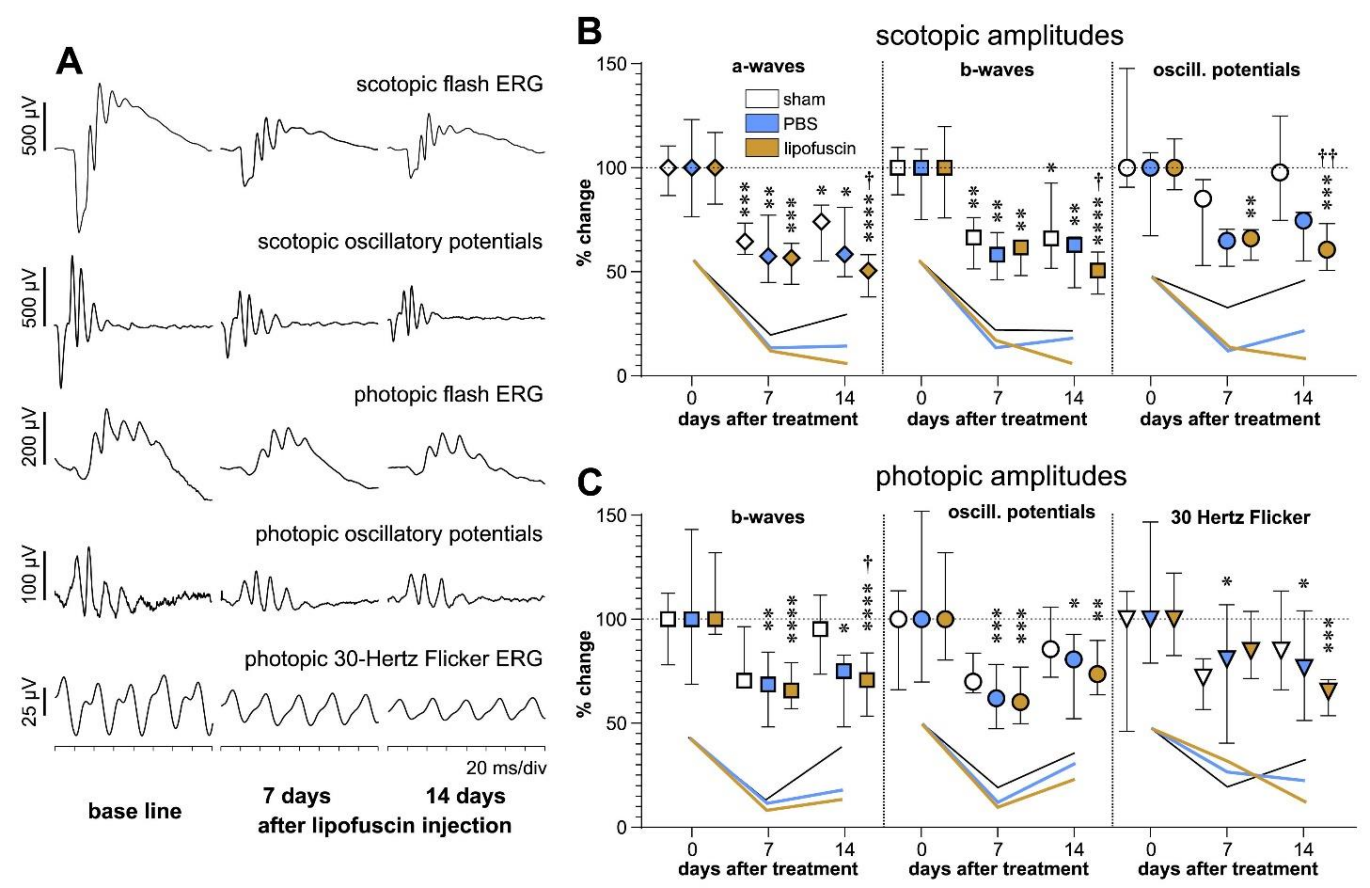
Functional testing by electroretinography. (A) Typical ERG waveforms obtained in the mice by different techniques indicated above the curves. In the example, results of ERG measurements are shown before injection and 7 days and 14 days after a subretinal injection of LF. (B) Changes of amplitudes of scotopic parameters and (C) changes of amplitudes of photopic parameters compared to baseline 7 days and 14 days after sham treatment (white symbols) or injection of PBS (blue symbols) or LF (dark yellow symbols). Median values with interquartile ranges are shown. Numbers of animals for sham, PBS and LF groups were before treatment n=6, n=14 and n=11, 7 days after injection n=6, n=7 and n=8, and 14 days after injection: n=5, n=8 and n=8, respectively. Symbols show amplitudes of different ERG parameters: ⋄ a-waves, ▯ b-waves, ◯ oscillatory potentials, ▽ 30 Hertz Flicker. Asterisks show statistical significance of differences compared to corresponding base line values: * p<0.05, ** p<0.01, *** p<0.001, **** p<0.0001, with p values calculated by the Kruskal-Wallis test with Dunn's multiple comparisons test. Crosses indicate significance of differences compared to corresponding values after sham treatment: † p<0.05, †† p<0.01.

Thickness of the retina and the RPE were determined in OCT scans. To obtain a better picture, OCT scan were taken at three different positions, the first straight through the optic nerve head, the second scan passing nearby the optic nerve head, and the third scan through the site of injection (Fig. 4). It can be seen that retinal thickness and RPE layer thickness remain constant after injection of PBS, whereas they both decrease clearly after injection of LF.

To check consequences of subretinal injection of LF on retinal function, we performed ERG measurements in both scotopic and photopic modes (Fig. 5). After injection, amplitudes of most ERG parameters decreased clearly. A decrease of amplitudes was also be observed after sham intervention, too, although not so sharply. Scotopic, rod-driven amplitudes that were decreased 7 days after sham treatment or PBS injection did not decrease further. Some of the parameters even showed a slight tendency of recovery after 14 days. In contrast, amplitudes of scotopic parameters after injection of LF decreased slightly after 14 days. There was no statistically significant difference between decreased amplitudes after PBS injection and LF injection or sham treatment after 7 days or 14 days. However, amplitudes of scotopic ERG responses were significantly more decreased after LF injection than after sham treatment after 14 days (crosses in Fig. 5).

In case of photopic, *i.e.*, cone-driven ERG, the decrease of amplitudes was not as impressive as in scotopic ERG. The recovery of amplitudes after sham treatment or PBS injection was more prominent in the photopic mode than in the scotopic mode. Again, amplitudes did not recover after injection of LF, except for a-wave amplitudes.

**Figure 6.**
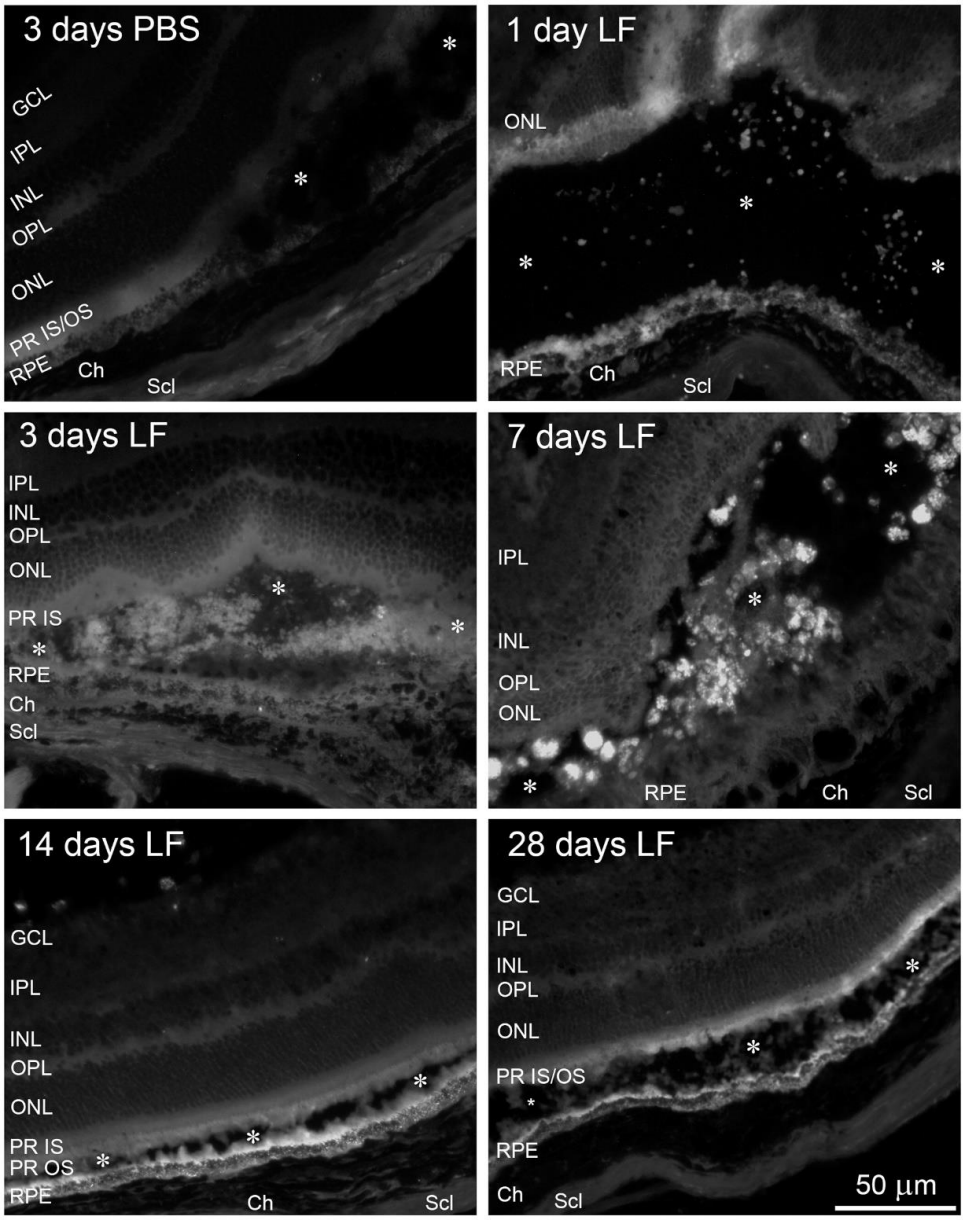
Distribution of lipofuscin (LF) in the subretinal space. Cryosections of mouse eyes to visualise lipofuscin (LF) by its autofluorescence at 488 nm 1, 3, 7, 14 and 28 days after injection as indicated. For comparison, a sample is shown obtained 3 days after injection of PBS. Asterisks indicate subretinal space. We checked cryosections of eyes of 5 animals for each time point, and typical results are shown.

We then isolated the eyes and prepared cryosections in order to check localisation of injected LF particles by their autofluorescence. One day after subretinal injection, it was difficult to find tiny LF particles that were distributed loosely in the subretinal space (Fig. 6). At later time points, we found aggregates of autofluorescent material, which were particularly clearly visible seven days after LF injection. 14 days or 28 days after injection of LF, these aggregates were gone, and a relatively uniform autofluorescence was visible in the subretinal space nearby the RPE and inside the RPE (Fig. 6).

**Figure 7.**
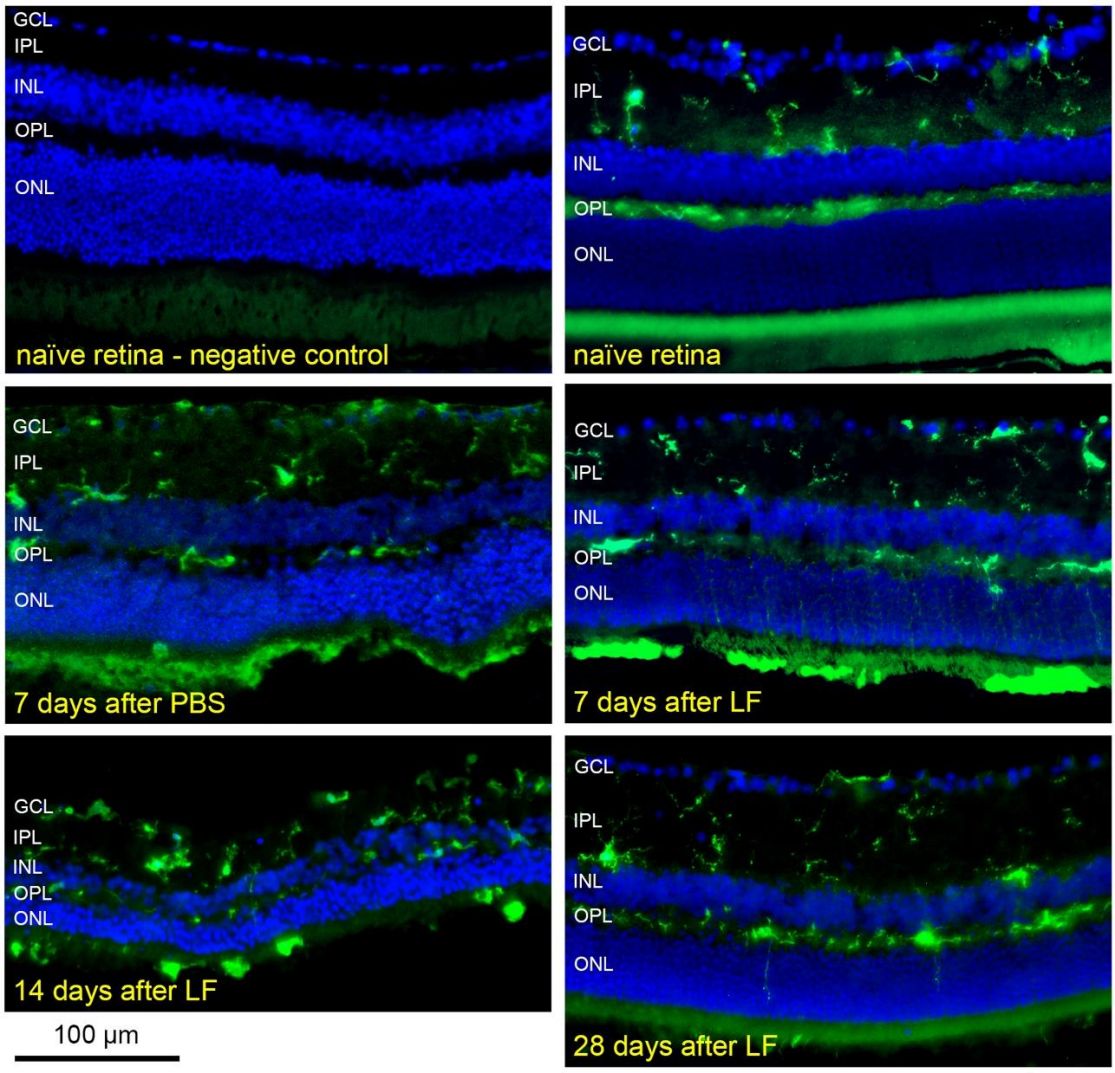
Distribution and morphology of microglial cells after injection of LF. Microglial cells positive for Iba1 in cryosections of a treatment naïve retina, 7 days after subretinal injection of PBS, or 7, 14 and 28 days after subretinal injection of LF as indicated. Many cells positive for Iba1 are located in the subretinal space 7 and 14 days after LF injection, and they display an amoeboid shape. We checked cryosections of eyes of 3 animals for day 7 and of 6 animals for the other time points and the controls, and typical results are shown.

The question was why LF gathered in such aggregates during the first week after subretinal injection. We hypothesised that LF particles could be engulfed by microglial cells, the resident immune cells in the retina, and that these aggregates are in fact phagocytic microglial cells filled with lipofuscin. For such a behaviour, the first prerequisite is activation of microglial cells and their migration from the inner retina to the subretinal space. In fact, we found numerous microglial cells in the outer retina 7 days and 14 days after an injection of LF (Fig. 7). In addition, microglial cells located in the outer retina display an irregular, enlarged shape, without processes, which is typical for their transition into an activated, macrophage-like state. 14 days after injection, the majority of microglial cells even in the inner retina displayed a shape typical for an activated state. Many thin green traces could be seen in the outer nuclear layer (ONL), in particular 7 days after injection of LF. This raises the question about the way microglial cells pass the ONL. Microglial cells have to find a path through the ONL, and they became very thin and long stretched due to the densely packed photoreceptor nuclei in the ONL. 28 days after injection of LF, almost all microglial cells had returned into the inner retina, where they regained their ramified shape (Fig. 7).

**Figure 8.**
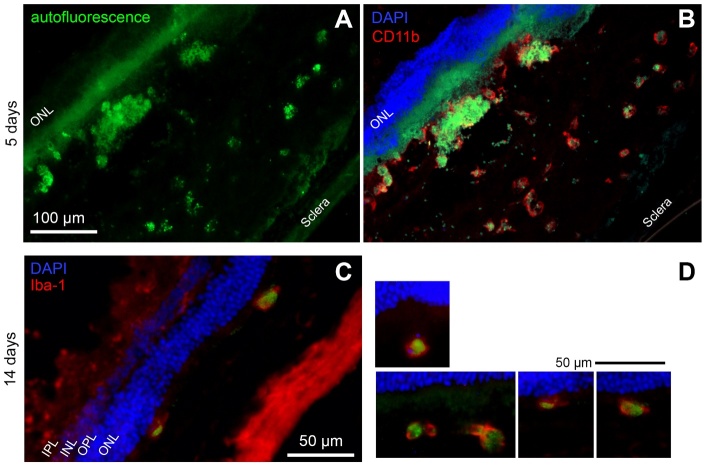
Microglial cells have phagocytosed LF. (A) Autofluorescence of a retinal cryosection five days after subretinal injection of LF. Aggregates of autofluorescent material in the subretinal space are clearly visible. (B) The same site of the sample, after immunostaining against CD11b using a red fluorescent dye. Co-localisation of LF autofluorescence and CD11b can be clearly seen. (C) Green LF autofluorescence can be seen in microglial cells labelled against Iba1 in the subretinal space 14 days after subretinal injection of LF. (D) More examples of Iba1-positive microglial cells in the subretinal space 14 days after LF injection. Nuclei stained with DAPI on top of the images belong to the outer nuclear layer. We checked cryosections of eyes of 5 animals for each time point, and typical results are shown.

To check if microglial cells located in the subretinal space really phagocytosed LF, we performed immune-labelling of microglia using a secondary antibody that shows fluorescence in the red light where LF does not show fluorescence. We could identify many roundish cells positive for CD11b or Iba1 that display green fluorescence in their cytoplasm, indicating that microglial cells indeed have engulfed LF (Fig. 8).

**Figure 9.**
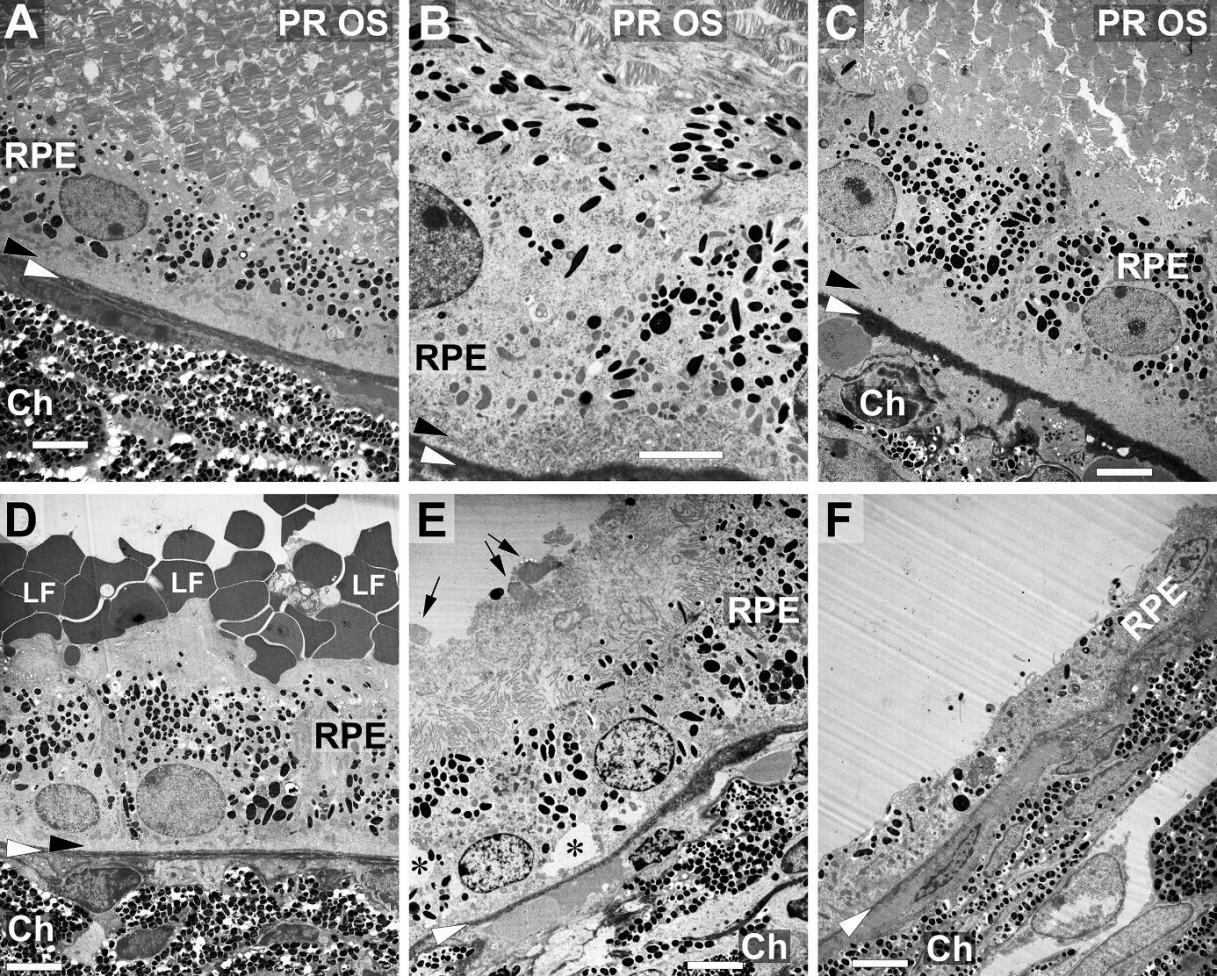
Changes of outer retina morphology on ultrastructural level. (A) Typical appearance of outer ocular structures of an untreated mouse eye in transmission electron microscopy including photoreceptor outer segments (PR OS), RPE cells and the choroid (Ch) with its densely packed melanocytes. Black arrowheads point to the basal labyrinth of the RPE cells, and white arrowheads to Bruch’s membrane in every image. Appearance of a corresponding area 7 days (B) or 21 days (C) after subretinal injection of PBS. Photoreceptor outer segments, RPE cells with the basal labyrinth and Bruch’s membrane look quite normal. (D) Seven days after subretinal injection of LF, many LF particles can be seen on the apical side of the RPE cells. (E) Structure of RPE cells shows signs of beginning deterioration 21 days after injection, with asterisks indicating vacuoles. Arrows point to some fragments of PR OS, and a lot of poorly defined debris can be seen on top of the RPE cells. Some RPE cells have almost degenerated 21 days after injection of LF (F). Ch: choroid. Scale bars: 5 µm in all images.

We also checked the morphology of the outer retina and RPE on ultrastructural level by transmission electron microscopy (TEM, Fig. 9). Morphology did not change notably 7 days (Fig. 9B) or 21 days (Fig. 9C) after sub-retinal injection of PBS compared to control retinas (Fig. 9A). Photoreceptor outer segments were intact and approached the RPE cells. All cellular structures did not show any signs of degeneration.

Subretinal injection of LF led to several morpho-logical changes in the RPE cells. Numerous grey particles were seen at many places on top of the RPE 7 days after injection, *i.e.*, on the apical side of the RPE cells (Fig. 9D). 21 days after injection, the structure of the RPE started to deteriorate (Fig. 9E), and a high number of microvilli was destroyed. Inside the RPE cells, many grey inclusions could be found besides black melanin granules. Moreover, empty “holes” could be seen, most probably vacuoles (asterisks). In addition, thickness of Bruch’s membrane (white arrowheads) showed variations. Basal labyrinth of the RPE cells (black arrowheads), which was thinner 7 days after LF injection, was lost 21 days after injection. In extreme cases, RPE cells started to degenerate (Fig. 9F). Thickness of RPE cell layer decreased clearly, and RPE cells lost most of melanin granules. Bruch’s membrane also showed clear signs of decomposition.

**Figure 10.**
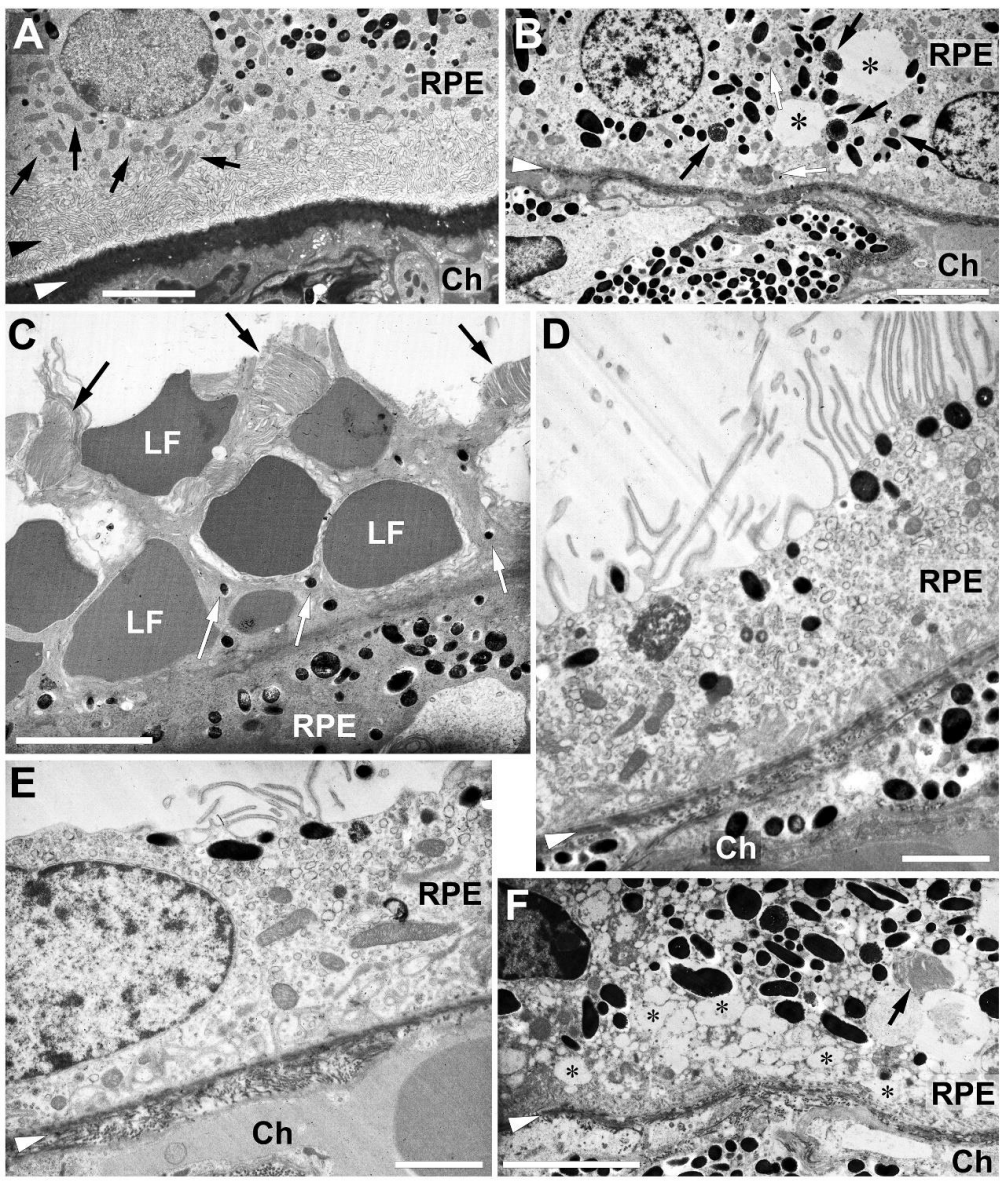
Ultrastructural details of the RPE. (A) Black arrowhead points to the basal labyrinth of the RPE cells, which is intact in control eyes. White arrowheads point to Bruch’s membrane in every image. Black arrows point to some of the mitochondria. (B) No labyrinth is left 21 days after injection of LF. Black arrows point to decomposing melanosomes, and white arrows to LF inclusions. (C) LF granules are intermingled with microvilli (white arrows) 7 days after LF injection. Black arrows point to fragments of photoreceptor outer segments (D) and remnants of microvilli 21 days after injection of LF (E). Bruch’s membrane shows signs of beginning decomposition (F). Big number of vacuoles inside an RPE cell (asterisks). Ch: choroid. Scale bars: 5 µm in (A), (B), (C) and (F), and 2 µm in (D) and (E).

Some more details are shown in Figure 10. Disappearance of the basal infoldings, the so-called basal labyrinth, after injection of PBS is demonstrated in Figs. 10A and 10B. Whereas a clear labyrinth can be seen in the RPE of a control eye (Fig. 10A), it is gone 21 days after injection of LF (Fig. 10B). Some melanosomes inside the RPE cells show signs of disintegration (black arrows), and grey inclusions are seen, most probably small lipofuscin granules (white arrows).

LF granules are visible 7 days after injection of LF on top of the RPE cells in Fig. 10C. They are intermingled with microvilli, and there are some melanin granules visible in the bunches of microvilli (white arrows). There are still some fragments of photoreceptor outer segments visible (black arrows). Microvilli are destroyed widely 21 days after LF injection (Figs. 10D and 10E). Bruch’s membrane displays variations in thickness, most probably due to decomposition (Fig. 10E). In some cases, vacuoles mentioned above after LF injection are visible in a high number in the RPE cells, giving the cytoplasm of the RPE cells a foam-like appearance (Fig. 10F).

**Figure 11.**
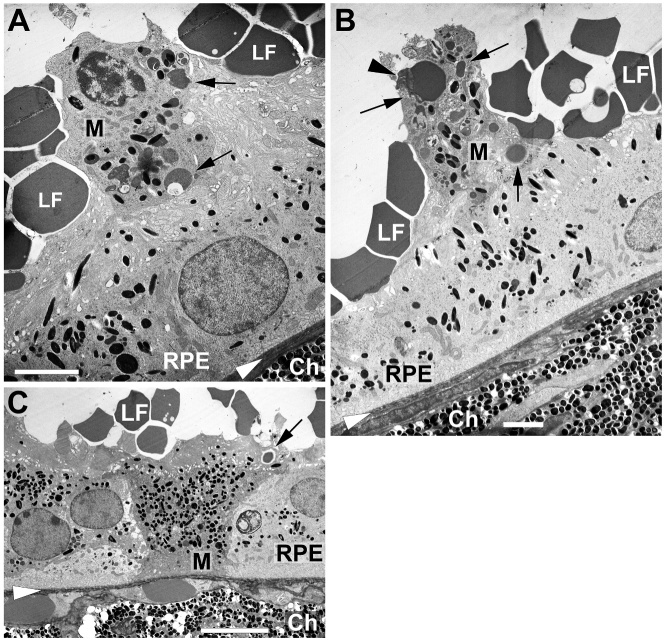
Macrophages loaded with LF and melanin nearby the RPE. Examples of macrophages (M) nearby the RPE cells 7 days after subretinal injection of lipofuscin (LF). LF particles are visible on top, on the apical side of RPE cells. (A+B) Macrophages on top of RPE cells. Arrowhead points to a fragment of a photoreceptor outer segment. (C) A macrophage between two RPE cells. Black arrows point to some large LF granules engulfed by macrophages. White arrowheads indicate position of Bruch’s membrane. Ch: choroid. Scale bars: 2 µm in (A) and (B), 5 µm in (C).

In Figure 8, the presence of macrophage-like microglial cells was demonstrated in the subretinal space that had phagocytosed autofluorescent LF. Such cells were also found in the TEM images (Fig. 11). They can be identified by their irregular shape and a slightly denser cytoplasm compared to RPE cells. Moreover, they have taken up several melanin granules, and more importantly, LF granules that display the same grey scale hue as the large LF granules on top of the RPE cell layer. Two of those macrophage-like cells are situated on top of the RPE cells (Figs. 11A and 11B), and one large cell with a particular high amount of melanin and LF granules was found inside the RPE cell layer, between the RPE cells (Fig. 11C).

**Figure 12.**
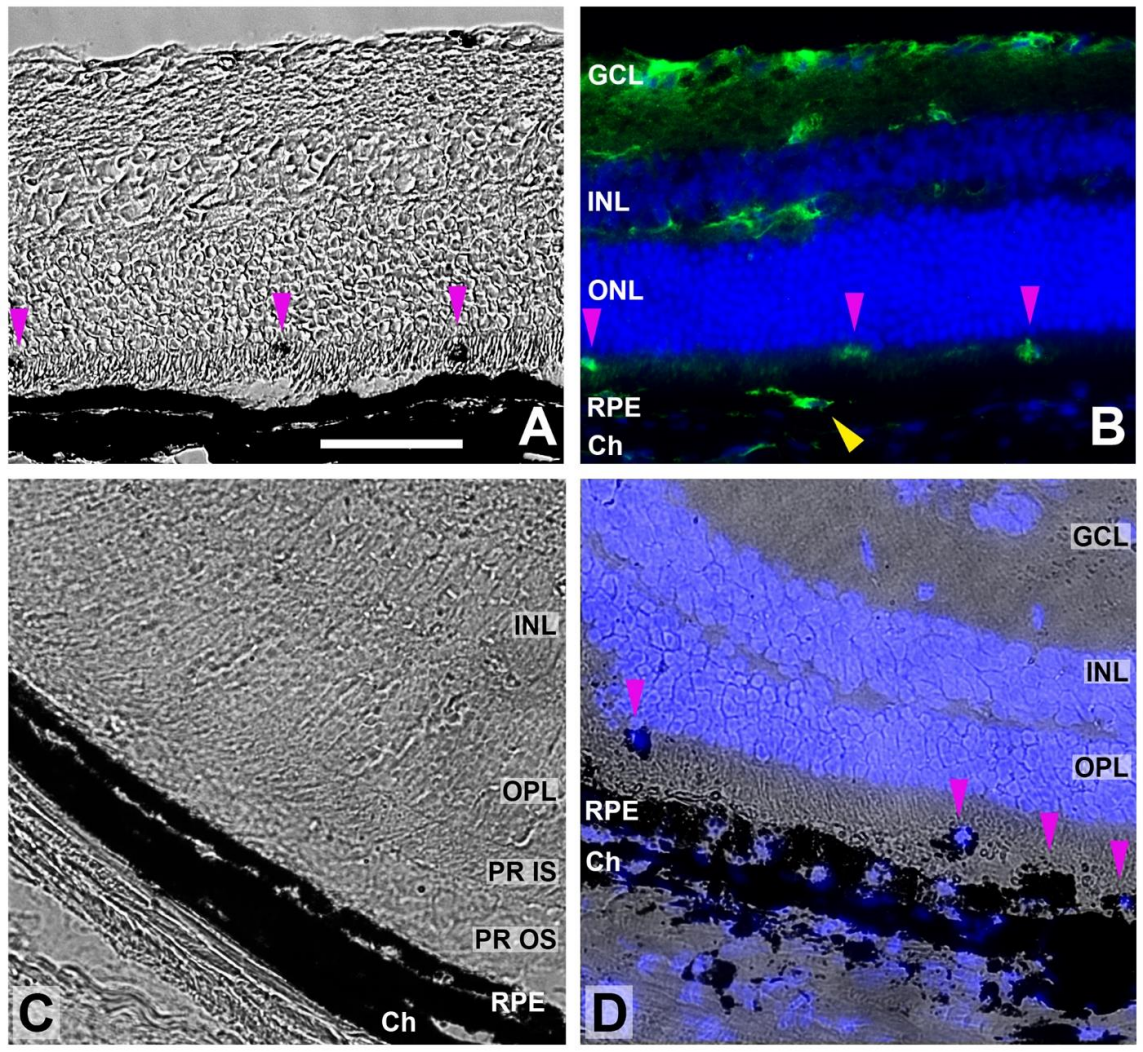
Melanin-loaded cells in the outer retina. (A) Transmitted light image of a retinal cryosection obtained 7 days after subretinal injection of LF. Pink arrowheads point to dark aggregates, probably cells filled with melanin. (B) The same sample with microglial cells stained for Iba1 (green) and cell nuclei with DAPI (blue). Dark cells visible in (A) are positive for Iba1 (pink arrowheads). Yellow arrowhead points to another microglial cells on top of the RPE cell layer that cannot be distinguished from the RPE in (A). (C) Image of a retinal cryosection obtained 21 days after subretinal injection of PBS. RPE cell layer and choroid (Ch) are preserved well. (D) Image of a retinal cryosection obtained 21 days after subretinal injection of LF. RPE cell layer and choroid look distorted, and RPE cells have lost part of their melanin. Cells containing melanin are visible in the subretinal space, and their nuclei display blue DAPI staining (pink arrowheads). Scale bar: 50 µm.

After finding macrophage-like cells filled with melanin, we again checked histological sections obtained after subretinal injection of LF (Fig. 12), and we found cells filled with dark material also in the subretinal space in frozen sections (Figs. 12A and 12D). They were positive for the microglial marker Iba1 (Fig. 12B). 21 days after an injection of PBS, the RPE cell layer and the choroid were not affected (Fig. 12C), whereas the morphology of these layers was clearly distorted 21 days after injection of LF (Fig. 12D).

**Figure 13.**
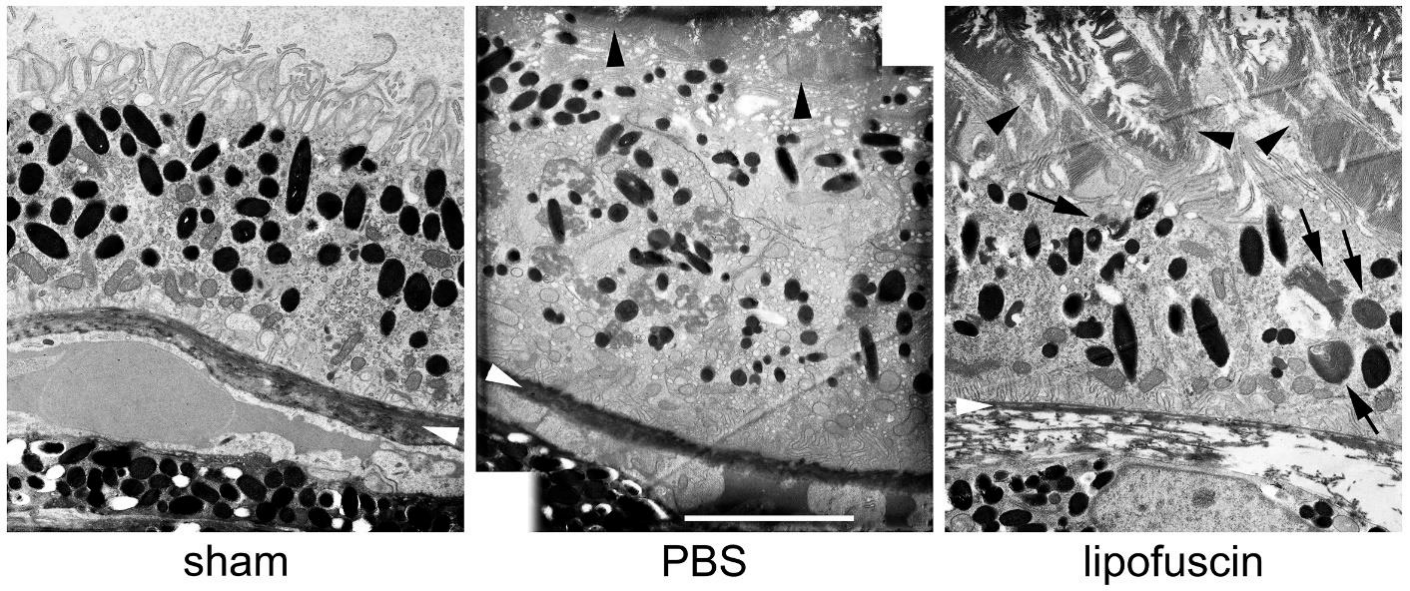
Ultrastructure 6 months after injections. EM images obtained 6 months after treatments as indicated. White arrowheads point to Bruch’s membrane. Black arrowheads point to photoreceptor outer segments. In the right image, black arrows point to some LF granules. Scale bar: 5 µm.

Differences between sham-treated eyes, eyes injected with PBS and eyes injected with LF were seen also six months after the treatments (Fig. 13). We found a reduced size of the basal labyrinth in the eyes of all three groups, as well as shortened microvilli on the apical side of the RPE. The general appearance of the RPE was normal in the sham-treated eyes and eyes injected with PBS, with normal melanin granules and mitochondria, although some smaller distortions were visible in the PBS group. Bruch’s membrane was normal in both groups. Eyes treated with LF showed a clear disintegration of the Bruch’s membrane, and only a very thin layer remained on most inspected sites, if at all. In contrast to eyes injected with PBS, a clear deterioration of the photo-receptor outer segments can be seen in the eyes injected with LF. Number of melanin granules is reduced, and several lipofuscin granules are present in the RPE cells.

**Figure 14.**
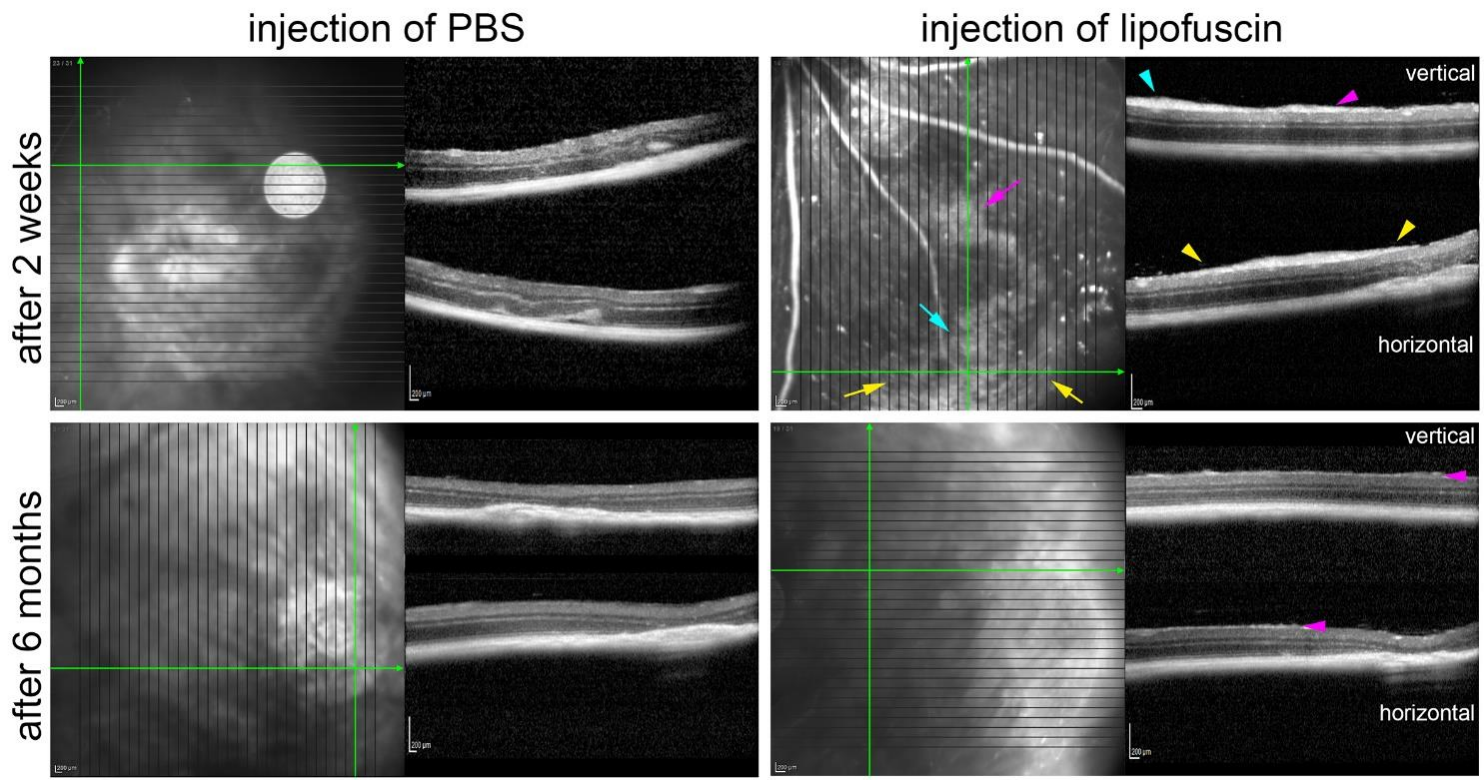
Epiretinal membranes after injection of LF. OCT images obtained two weeks and 6 months after treatment as indicated. Two weeks after injection of LF, clear epiretinal membranes are visible. Coloured arrows in the SLO image point to diffuse reflective areas, and arrowheads in the OCT sections with the same colour point to corresponding sites where the epiretinal membranes are visible. Six months after LF injection, only very tiny structures are visible on top of the retina (magenta arrowheads). No hint on any epiretinal membranes is visible after subretinal injection of PBS.

**Figure 15.**
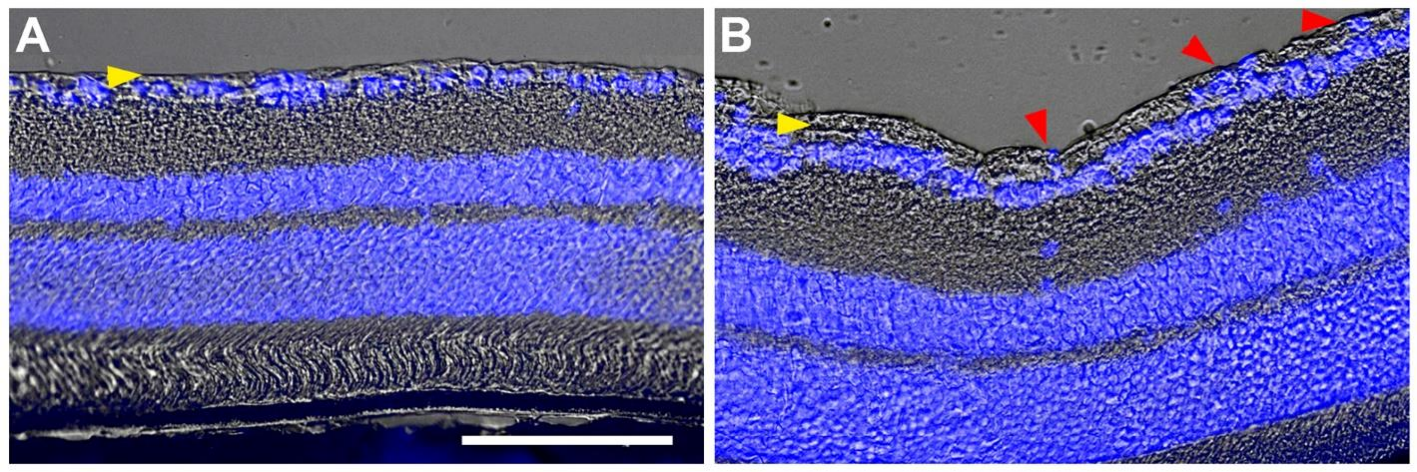
Epiretinal membranes after injection of LF. Histological sections of retinas 35 days after subretinal injection of PBS (A) or LF (B). Yellow arrowheads point to the nerve fibre layer. After injection of LF, there is a thickening of this layer, and blue DAPI staining indicates presence of cell nuclei in this layer. Scale bar: 100 µm.

Another finding was a transient formation of epi-retinal membranes in the eyes injected with LF. They were found in almost all eyes 14 days after subretinal injection of LF in the OCT images (Fig. 14). Six months after subretinal injection of LF, the epiretinal membranes were not visible clearly anymore (Fig. 14). In some cases, we found hints for epiretinal membranes after injection of LF also in histological sections, were DAPI-stained nuclei suggested a cellular nature of the thickened layer (Fig.15).

**Figure 16.**
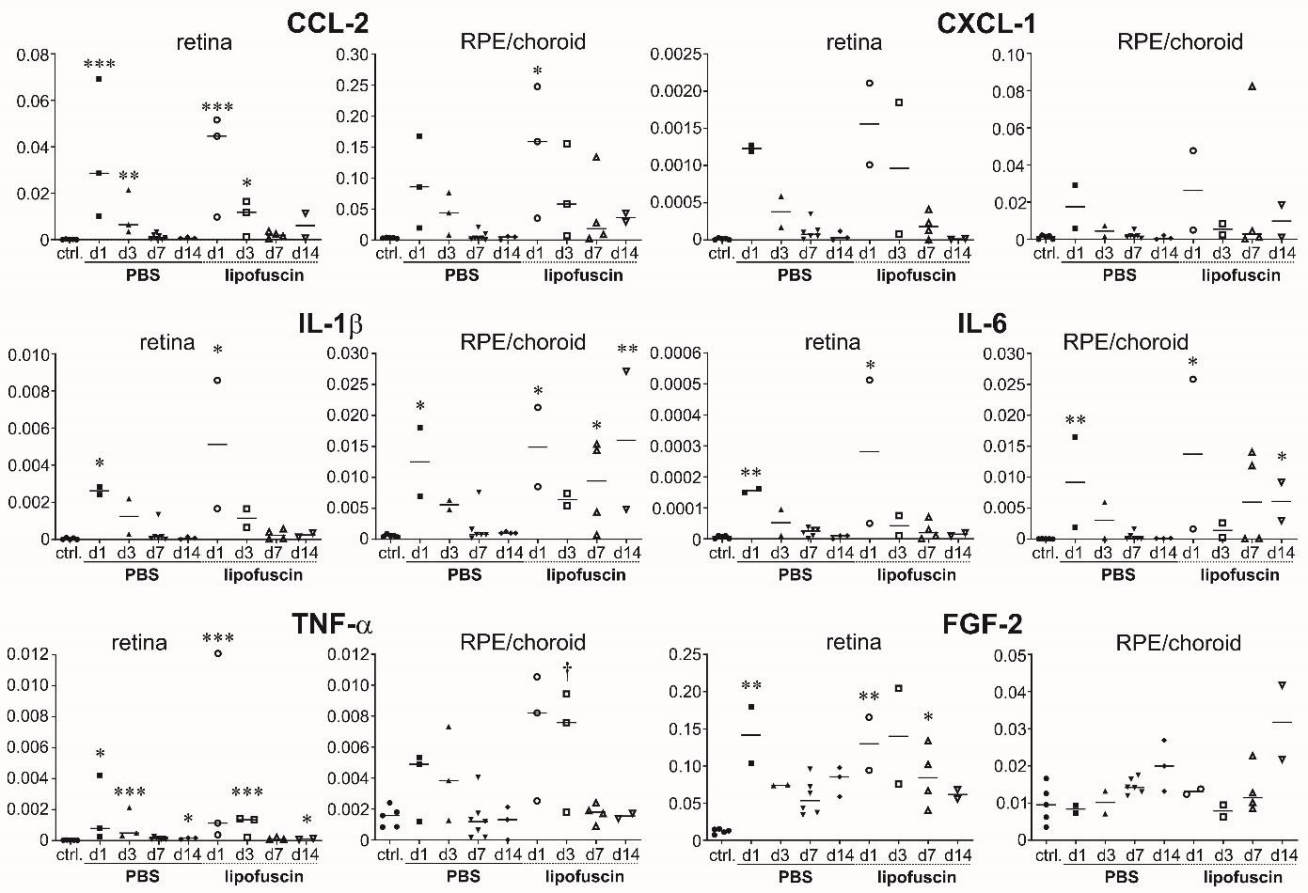
Levels of mRNA of inflammatory cytokines are increased after injections. Results of quantitative PCR of different inflammatory cytokines (CCL-2, CXCL-1, IL-1β, IL-6, TNF-α) and the growth factor FGF-2 as indicated above the diagrams in the non-treated eye (control) and 1, 3, 7 and 14 days after subretinal injection of PBS or LF. Retina and RPE/choroid complex were analysed separately. Between 2 and 6 samples were analysed per time point. In the dot blots, horizontal lines represent the median values of the data. Note the different scales. Significance of differences between controls and treated eyes are shown by asterisks: * p<0.05, ** p<0.01, *** p<0.001, with p values calculated by the Kruskal-Wallis test with Dunn's multiple comparisons test. † shows significant difference between injection of PBS and injection of LF (p<0.05).

We finally checked expression of some pro-inflammatory cytokines and growth factors. Quantitative PCR analysis was performed separately for the retina and the RPE/choroid complex (Fig. 16). As a general finding, it can be stated that there is an increase in the amount of the mRNA of the inflammatory cytokines CCL-2, CXCL-1, IL-1β, IL-6 and TNF-α on the first days after subretinal injection of both PBS and LF in the retina as well as in the RPE/choroid complex. There was no statistically significant difference between the values obtained after injection of PBS or LF, nevertheless increase was slightly higher after injection of LF than after injection of PBS. 7 days or 14 days after injection, mRNA levels in the retina returned to control levels except for CCL-2. In the RPE/choroid complex, mRNA levels of inflammatory cytokines also decreased to control levels 14 days after subretinal injection of PBS. In contrast, they did not decrease to control levels after subretinal injection of LF, in particular for IL-1β and IL-6. Levels of mRNA of inflammatory cytokines were clearly much higher in the RPE/choroid complex than in the retina.

The levels of mRNA of the growth factor FGF-2 showed a different behaviour after subretinal injections (Fig. 16). In the retina, they first increased and then remained on a level higher than in the controls, whereas in the RPE/choroid complex they were similar to control levels 1 day and 3 days after injections and were increased 7 days and 14 days after injections of PBS or LF. Levels of FGF-2 mRNA were much higher in the retina than in the RPE/choroid complex, whereas mRNA levels of VEGF-A did not show such differences.

## DISCUSSION

Decline of vision in the course of aging is connected with a substantial loss of quality of life. The situation gets even worse when vision is additionally affected by age-related macular degeneration, which can lead to functional blindness in its advanced stage. Lipofuscin (LF), the so-called “age pigment”, may play a role in intermediate and advanced age-related macular degeneration (AMD). We therefore sought to investigate the processes in the back of the eye, around the retinal pigment epithelium (RPE), in the presence of LF. For this purpose, we injected a suspension of LF from human donor eyes subretinally into the eyes of mice. We found a strong microglial response. Microglial cells migrated to the subretinal space and phagocytosed a big portion of the injected LF within a few days. RPE cells also took up a part of the LF particles. While the microglial cells disappeared from the subretinal space, most probably by migration back to the inner retina, RPE cells showed signs of degradation and even started to degenerate.

To study pathomechanisms and potential treatment options of AMD, animal models are highly desirable. The mouse is a commonly used animal in retinal research, as the structure of the retinal layers is quite similar in most mammals, except for the macula with the fovea that is present just in primates. Moreover, retinal physiology and most of the factors and signalling pathways are very similar in mice and humans. For neovascular AMD, the experimental model of laser-induced choroidal neo-vascularisation in the mouse has been used broadly for many years, despite its limitations [12,13]. Several animal models have been proposed for intermediate AMD or geographic atrophy over the past years, based on environmental, metabolic or genetic factors that are known to promote AMD, such as the “cigarette smoke” model, damage by light, oxidative damage by carboxyethylpyrrole or deactivation of antioxidative mechanisms, knock-out of chemokine or complement factor genes, dysregulation of lipid metabolism, and others (for reviews, see [14-19]). Although these models do not show all facets of the disease in patients, they provided valuable insights into the mechanisms of retinal degeneration.

Due to the short life span of mice, LF does not accumulate in the RPE of a normal healthy mouse to an extent that is sufficient to cause any processes related to intermediate or advanced AMD. Therefore, we tried to elicit such processes by a subretinal injection of LF isolated from human donor eyes. Success of subretinal injections was checked by OCT, by which retinal detachment could be visualised.

Functional testing by ERG measurements revealed a clear decrease of retinal function after injection, no matter if PBS or LF suspension were injected, and to our surprise, even after sham treatment. One of the authors detected such a decrease also in other cases of intraocular injections, *e.g.* after intravitreal injections, and it may take a longer time until the retinal function is recovered [20]. Therefore, we checked by how much ERG amplitudes recovered after subretinal injections. Recovery was the best after sham treatment, and photopic ERG amplitudes recovered better than scotopic ERG amplitudes.

We did not find differences in the amplitudes between PBS and LF groups 7 days after injection, and amplitudes were also not significantly different from the sham group. Significant difference was found, however, 14 days after injections between scotopic ERG amplitudes of the sham group and the LF group, indicating that LF injection may have caused deterioration especially of rod function. Gresh *et al.* (2003) reported about declined function of rod photoreceptors in aged mice, whereas short-wavelength cones did not show such a loss, which corresponds to our findings of a bigger loss of rod function and a better recovery of cone function [21]. Moreover, rods lost their function in particular in albino mice [21], which goes in line with our finding that RPE cells lose melanin after injection of LF. Kolesnikov *et al.* (2010) also described in more detail that rods lose function with age, whereas function of cones remained more stable [22].

Due to autofluorescent properties of LF at shorter wavelengths [23], we anticipated it could be easily detected in histological sections. Indeed, it could be seen in cryosections at different time points after subretinal injection using a 488 nm/525 nm filter (Fig. 6). While LF showed an even distribution in tiny dots 1 day after injection, an accumulation in aggregates was seen 7 days after injection. The reason for such aggregates became clear when the behaviour of microglial cells was checked. After subretinal injection of LF, we saw numerous microglial cells migrate through the outer nuclear layer (ONL) into the subretinal space, where they remained some days (Fig. 7). Presence of microglial cells in the subretinal space, in the vicinity of the RPE cell layer, is a common feature of AMD in patients and experimental models [6-9,24]. In our study, microglial cells turned into a macrophage-like phenotype and phagocytosed quickly LF granules, as could be demonstrated by labelling for microglial markers (Figs. 8, 12B). RPE cells lost their melanin, and microglial cells took it up, as can be seen in Fig. 12. Notably, macrophage-like cells were seen also in TEM images, filled with small LF and melanin particles (Fig.11).

**Figure 17.**
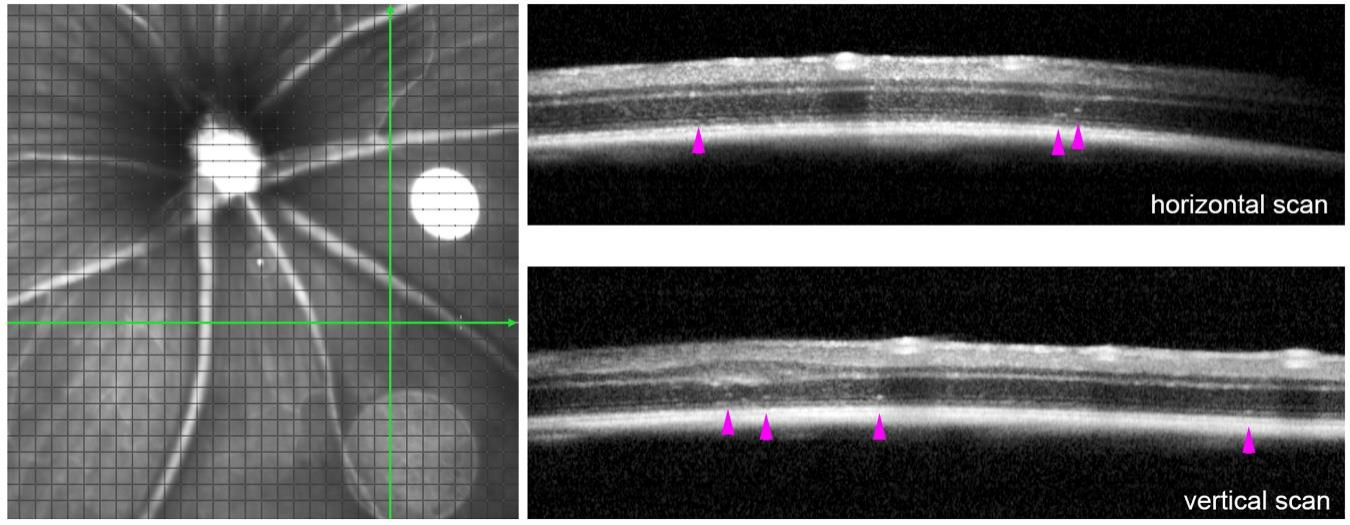
Hyperreflective foci after injection of LF. OCT scans of the eye whose histological image is shown in Fig. 12D. Pink arrowheads point to bright spots possibly equivalent to hyperreflective foci.

These findings and the presence of macrophage-like cells nearby the RPE visible in the EM images are an indication that retinal microglial cells that have phagocytosed material from damaged RPE cells are present in the outer retina. In this context, we saw bright spots in the OCT images of the eye of the mouse whose histological image is shown in Fig. 12D (see Fig. 17). Such dots were not seen in OCT scans of eyes after injection of PBS. It may be speculated that these bright spots may be the equivalent to hyperreflective foci (HRF) seen in patients with intermediate AMD, and that the HRF in patients also represent such microglial cells.

It is not clear presently what happens to LF, and melanin engulfed by microglial cells. As noted above, microglial cells return to the inner retinal layers. It may be hypothesised that the microglial cells release phago-cytosed material into the blood circulation (see, *e.g.*, Fig. 5 in [25]), however, such a mechanism has yet to be proven in our model.

A small part of the injected LF was taken up by RPE cells, as can be deduced from the faint autofluorescence RPE cells show 14 days and 28 days after subretinal injection of LF (Fig. 6) and from the presence of grey particles in the RPE cells visible in the TEM images (Figs. 9D and 9E, and Fig. 13).

Ramkumar *et al.* (2010) reviewed ultrastructural changes in a big variety of animal models of “dry” AMD [16]. We compared the changes found in these experi-mental models with the changes we found in our mice after subretinal injection of LF. Damaged and/or lost apical microvilli were also found in SAMP8, Nr2e3^rd7^, and arrd2/arrd2 mice. In many of the cited mouse models, hypopigmentation of the RPE, *i.e.*, loss of melanin, was described (Ccr2^-/-^, Ccl2^-/-^/Cx3cr1^-/-^, Sod2 knockdown, mcd/mcd, Cp^-/-^/Heph^-/Y^, ApoE4 TR and arrd2/arrd2 mice). Degradation of basal infoldings of the RPE cells (the so-called “labyrinth”) was found in Efemp1^R345W/R345W^, Ccr2^-/-^ and Sod2 knockdown mice. We have also seen vacuolisation in the RPE cells, which was found in abcr^-/-^, Efemp1^R345W/R345W^, Ccl2^-/-^, Ccl2^-/-^/Cx3cr1^-/-^, Sod1^-/-^, Sod2 knockdown, neprilysin^-/-^, ApoE4 TR and CEP-immunized mice. Recently, a number of ultrastructural changes was found in the RPE and Bruch’s membrane of the 5XFAD mouse, an animal model for Alzheimer’s disease, which resemble features of “dry” AMD in patients, and that were found also in our animals (loss of basal labyrinth and apical microvilli, increased thickness of Bruch’s membrane, and LF granules inside the RPE cells [26]. These examples show that our mouse model resembles many features of other experimental mouse models for AMD.

We could not demonstrate unambiguously presence of basal laminar deposits that are reported in several animal models (5XFAD, Efemp1^R345W/R345W^, Sod2 knock-down, neprilysin^-/-^, mcd/mcd, ApoE4 TR, APO*E3 Leiden, APO B100, CEP-immunized and SAMP8 mice) as well as in the eyes of AMD patients [27,28].

The described changes after subretinal injection of LF happened already after less than a month, whereas it took several months or even more than a year until they were visible in the animal models summarised in [16] or the 5XFAD mouse.

Another model to mimic pathological changes of AMD in small rodents was to inject sodium iodate (NaIO_3_), subretinally [29] or into the systemic circulation [30]. After subretinal injection, NaIO3 causes a strong oxidative stress, and a quick degeneration of the RPE, adjacent photoreceptors and underlying choriocapillaris was observed [29]. After systemic application, deleterious effects on retinal structure and function became visible after a longer time, detected by OCT imaging and ERG measurements, depending on the applied dose of NaIO_3_ [30].

It remains to be clarified by which mechanism microglial cells are attracted to the subretinal space after injection of LF. Most probably, this could happen by soluble chemokines, such as CCL-2 and CXCL-1, and other inflammatory cytokines that are both expressed after subretinal injection. Elevation of their mRNA levels was also seen after injection of PBS, in both the retina and the RPE/choroid complex (Fig.12). Nevertheless, we saw a much smaller number of microglial cells migrating into the subretinal space after injection of PBS (not shown).

Formation of epiretinal membranes after injection of LF was an unexpected finding. It is not clear at the moment what kind of cells contributes to these membranes, and why they disappeared after six months to a big portion. Although it is known that epiretinal membranes occur predominantly in older eyes or after surgical interventions [31], and there are reports about epiretinal membranes in patients with geographic atrophy [32], no causal connection between AMD and such membranes is known so far.

A limitation of our study is that the detailed composition of the injected LF suspension is not known. LF consists of a big variety of oxidised and crosslinked lipids and protein fragments, and we did not characterise this mixture in detail. Moreover, we injected just one single dose of LF in this pilot study, as a proof of principle, if we can detect deleterious effects of LF at all. Subretinal injection always causes a certain damage to the retina, no matter which liquid is injected. Therefore, consequences of mechanical stress caused by subretinal injection may interfere with effects of the presence of LF in the subretinal space, which should be clarified in future studies. As subretinally injected LF distributed only over a small part of the back of the eye, it may not be surprising that we did not see bigger differences to PBS injection in the ERG measurements and the qPCR analysis. In further studies, possible changes in ERG parameters should be monitored of a longer duration, such as it was done by Koster *et al.* [30].

As a conclusion, we can state that we see some features of intermediate AMD or beginning geographic atrophy after a subretinal injection of LF. Moreover, several morphological changes found in our study are the same as described in various other animal models of “dry” AMD. These features and changes are:

1. RPE cells lose melanin and their structural integrity (loss of microvilli and the labyrinth), and eventually degenerate.
2. Immune cells (most probably microglia) invade the subretinal space.
3. Bruch’s membrane gets thicker and deteriorates later on.
4. Photoreceptors lose their structural integrity and degenerate.

We did not see drusen or drusenoid deposits, most probably due to our more acute approach, whereas it takes years to form drusen in humans. We consider it a major advantage of our model that we expose the cells in the subretinal space, in particular the RPE, with LF, which is a natural player in the emergence of AMD. Further research will be needed to characterise the processes in more detail when the retina and the RPE are confronted with LF.
